# Effect of hepatic steatosis on the progression of chronic hepatitis B: A prospective cohort and *in vitro* study

**DOI:** 10.18632/oncotarget.17380

**Published:** 2017-04-24

**Authors:** Yangqin Chen, Chunlei Fan, Yuhan Chen, Hui Liu, Shanshan Wang, Peiling Dong, Lei Li, Huiguo Ding

**Affiliations:** ^1^ Department of Gastroenterology and Hepatology, Beijing You’an Hospital Affiliated with Capital Medical University, Beijing 100069, China; ^2^ Department of Pathology, Beijing You’an Hospital Affiliated with Capital Medical University, Beijing 100069, China; ^3^ Beijing Institute of Hepatology, Beijing 100069, China

**Keywords:** hepatic steatosis, chronic hepatitis B, hepatocellular carcinoma cells

## Abstract

**Aim:**

To characterize the effect of hepatic steatosis (HS) on the progression of chronic hepatitis B.

**Methods:**

A total of 162 chronic hepatitis B (CHB) patients confirmed by liver biopsy were involved in this study. All subjects were prospectively followed-up for 5 years in real-life clinical practice. Fibrosis stage was determined using aspartate aminotransferase-to-platelet ratio index (APRI). The end-point was cirrhosis, liver cancer or death. The effects of steatosis on the biological behavior of hepatocellular carcinoma cells were investigated using oleic acid-induced lipid accumulation in HepG_2_, HLE, PLC, and SMMC-7721 cells.

**Results:**

Mean age, body mass index, and serum cholesterol were significantly higher in CHB patients with HS than those without HS at baseline (*p*< 0.05). The APRI was lower in patients without HS at baseline (*p*<0.05). Compared to patients with HS, APRI of patients without HS decreased significantly during the follow-up period (*p*<0.05). The 5-year cumulative incidence of cirrhosis were 4.17% and 5.19% in patients without and with HS, respectively (*p*>0.05). The multivariate analysis showed that older (RR 1.07, 95% CI 0.996-1.149, *p* = 0.065) and S3 stage of liver fibrosis (RR 3.50, 95% CI 0.812–15.117, *p*=0.093) were risk factors for the progression to cirrhosis. *In vitro*, cell steatosis promoted proliferation and migration of HCC cells and conferred cell cycle at S phase.

**Conclusion:**

The older and S3 stage of fibrosis may be risk factors for progression to cirrhosis in CHB patients with HS. HS may aggravate liver disease, promoting HCC progression.

## INTRODUCTION

Chronic hepatitis B (CHB) is a major etiology of cirrhosis and hepatocellular carcinoma (HCC) worldwide, especially in China [1]. The risk factors for the development of cirrhosis in CHB patients have been identified as advanced age, male sex, hepatitis B virus (HBV) DNA load, and viral mutations [2–6]. During the last few decades, nonalcoholic fatty liver disease (NAFLD) has emerged as the most common chronic liver disease in the general population worldwide. The prevalence of NAFLD ranges from 6% to 33% among those with CHB [7]. NAFLD encompasses a spectrum of liver manifestations, ranging from simple hepatic steatosis (HS) to nonalcoholic steatohepatitis, fibrosis, and cirrhosis, which may ultimately progress to HCC. At present, the prevalence of HS in CHB patients reportedly ranges from 13% to 33% [8–10]. Accordingly, with the increasing incidence of NAFLD, the number of patients with concomitant CHB and NAFLD continues to increase rapidly. However, the progression and antiviral outcomes of patients with CHB and HS remain unclear [11, 12]. In this study, we aimed to characterize the effects of HS on the progression of CHB and antiviral response in a prospective cohort. As the limitation of the short time of follow-up and the small number of case, we also evaluated the effect of steatosis induced by oleic acid (OA) on the biological behavior of HCC cells *in vitro*.

## RESULTS

### Patient baseline characteristics

A total of 162 patients were enrolled in this study. The demographic, biochemical, virological, and histopathological features of CHB patients with and without HS at baseline are summarized in Table 1. Patients with HS were older (*p* = 0.034) and had higher BMI (*p* = 0.000) and higher serum cholesterol (*p* = 0.003). No significant differences in sex, HBV DNA level, inflammation grade or fibrosis stage were observed between the two groups at the baseline. During follow-up, 133 (82.1%) patients received antiviral treatment with IFN and/or NAs. The NAs included entecavir (ETV), adefovir (ADV), lamivudine (LAM) and telbivudine (LDT).

**Table 1:** Comparison of demographic, biochemical, virological and histopathological characteristics between CHB patients with and without HS

	Steatosis (+)	Steatosis (-)	p-value*
	(n = 77)	(n = 85)	
Age, years	35.27 ± 9.43	32.12 ± 9.35	0.034
Sex, male, n	59	60	0.385
female, n	18	25	
BMI, kg/m^2^	24.07 ± 2.23	22.63 ± 2.22	0.000
HBV DNA			0.072
positive, n (%)	51 (66.23%)	67 (78.8%)	
negative, n (%)	26 (33.77%)	18 (21.2%)	
ALT, U/L	91.84 ± 107.73	114.00 ± 91.93	0.538
AST, U/L	52.26 ± 49.50	62.47 ± 58.53	0.232
GGT, IU/L	54.75 ± 46.31	58.31 ± 68.08	0.093
ALP, IU/L	89.73 ± 25.78	94.03 ± 37.58	0.665
Cholesterol, mmol/L	4.40 ± 1.08	4.05 ± 0.77	0.003
Inflammation (G)			
0	1 (1.30%)	0 (0)	0.152
1	33 (42.85%)	28 (32.9%)	
2	29 (37.67%)	37 (43.5%)	
3	12 (15.58%)	19 (22.4%)	
4	2 (2.60%)	1 (1.2%)	
Fibrosis (S)			0.270
0	8 (10.39%)	4 (4.71%)	
1	32 (41.56%)	33 (38.82%)	
2	21 (27.27%)	29 (34.12%)	
3	16 (20.78%)	19 (22.35%)	
Hepatic steatosis			
Mild	59 (76.62%)	ND	
Moderate	16 (20.77%)	ND	
Severe	2 (2.61%)	ND	

CHB, chronic hepatits B; HS, hepatic steatosis; BMI, body mass index; ALT, alanine aminotransferase; AST, aspartate aminotransferase; ALP, alkaline phosphatase; GGT, gamma-glutamyl transpeptidase

### Noninvasive evaluation of liver fibrosis

In this cohort of 162 patients, cirrhosis developed in 8 (4.9%) patients during the follow-up period, including 4 (5.19%) patients with HS and 4(4.71%) without HS. No statistical difference was determined in the cumulative probability of progression to cirrhosis in patients with or without HS (*p* = 0.869; Figure 2A). Compared with their baseline, the APRI of both patients with HS and without HS had all decreased during the follow-up period (*p*< 0.05). However, the APRI of patients without HS decreased more than those with HS (*p*<0.05; Figure 3A).

**Figure 1:**
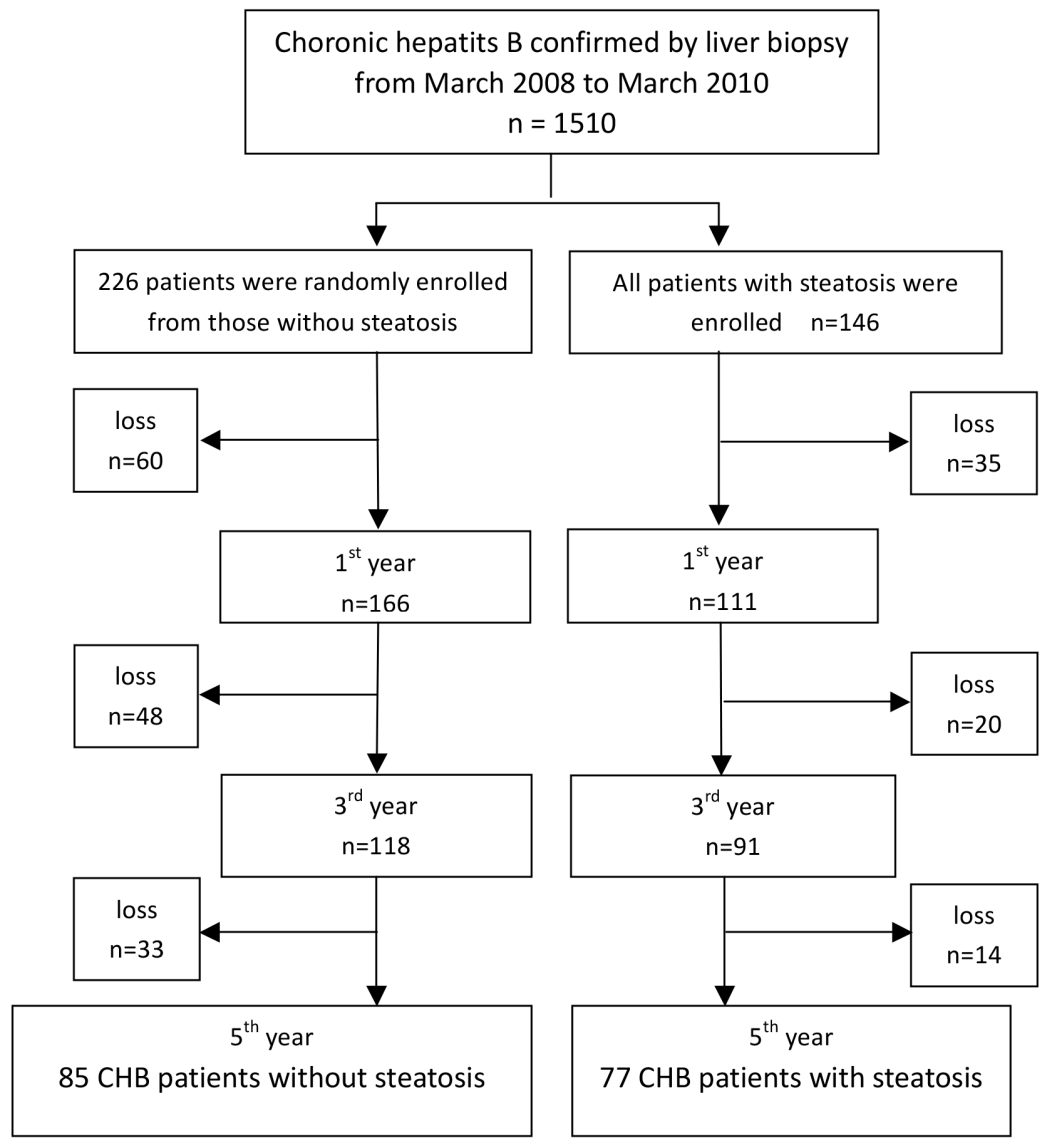
Flow chart of enrolment of chronic hepatitis B patients with or without steatosis

**Figure 2:**
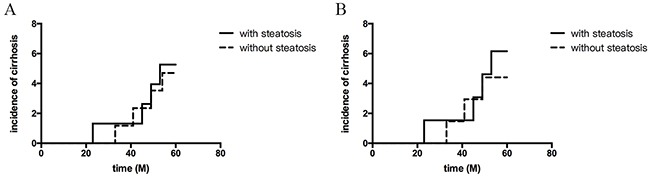
Cumulative probability of developing cirrhosis based on with or without hepatic steatosis **(A)** All patients during the follow-up period (by Kaplan–Meier method; log-rank, P>0.05); **(B)** Patients who received antiviral therapy (by Kaplan–Meier method; log-rank, P>0.05).

**Figure 3:**
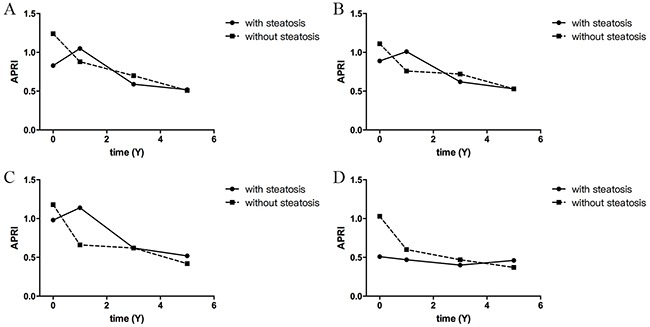
The trends of APRI during the follow-up period **(A)** All patients during the follow-up period. The APRI of both patients with and without HS had decreased significantly when they were compared to the baseline (patients with HS: *P*= 0.344, 0.013, 0.0021 in 1,3,5-year, respectively; without HS: all *P*<0.001, in 1, 3, 5-year). The APRI of patients without HS had decreased more than that of those with HS (P<0.05, by repeated measures analysis of variance). **(B)** Patients who received antiviral therapy and **(C)** patients who achieved CVR. The APRI of both patients with and without HS had decreased when they were compared to the baseline(P<0.05). However, the difference between patients with HS and without was not significant (P>0.05, by repeated measures analysis of variance). **(D)** Patients without antiviral therapy. In patients without antiviral therapy, the APRI of both patients with and without HS had not changed significantly (P>0.05), and the difference between them was not significant (P>0.05 by repeated measures analysis of variance). (APRI: AST-to-PLT ratio index; CVR, complete virological response)

### Evaluation of antiviral therapy outcome

A total of 104 patients achieved a complete virological response (CVR) and 29 patients failed to CVR or relapsed during the follow-up period. The presence of HS did not seem to affect the outcome of antiviral therapy (*p* = 0.942, Table 2). In patients who received antiviral therapy, the cumulative probability of progression to cirrhosis was not significant between patients with and without HS (*p* = 0.660, Figure 2B). In patients who received antiviral therapy, the APRI of both patients with HS and without HS had all decreased when compared to the baseline (*p*< 0.05), and no statistical difference was found between patients with or without HS (*p*>0.05, Figure 3B). Between patients with and without HS who achieved CVR, the decreased APRI was not significantly during the follow-up period (*p*>0.05, Figure 3C). In patients without antiviral therapy, the APRI of both patients with and without HS had not changed significantly (*p*>0.05, Figure 3D), and the difference between them was not significant (*p*>0.05).

**Table 2:** The response of antiviral therapy of CHB patients with or without HS

Treatment	Steatosis (+) (n, %)	Steatosis (-) (n, %)
Without antiviral therapy	12 (15.6%)	17 (20%)
Failed to respond or relapsed	14 (18.2%)	15 (17.6%)
CVR	51 (66.2%)	53 (62.4%)
IFN+NAs	15	14
IFN	5	4
ETV	13	13
LdT	2	5
LAM	3	2
ADV	9	7
NAs combination	4	8

CVR, complete virological response; IFN, pegylated interferon; ETV, entecavir; ADV, adefovir; LAM, lamivudine; LdT, telbivudine; NAs, nucleotide/nucleoside analogues

### Risk factor analysis

The multivariate analysis showed that patients who developed to cirrhosis were older (RR 1.07, 95% CI 0.996-1.149, *p*= 0.065) and had S3 stages of liver fibrosis (RR 3.50, 95% CI 0.812–15.117, *p*= 0.093). No significant effects of sex, BMI, HBV DNA level, HS, or higher grade of inflammation development on progression to cirrhosis were also observed by univariate analysis (Table 3).

**Table 3:** Univariate analysis of risk factors for the progression to cirrhosis

Factor	Cirrhosis (+)	Cirrhosis (-)	*p*-value
	(n = 8)	(n = 154)	
Age	40.13±12.32	32.25±9.23	0.045
Males/females, n (%)	5/3 (62.5%)	115/39 (74.67)	0.436
BMI	22.99±1.34	23.33±2.38	0.688
Family history of HBV (yes/no)	2/6	63/91	0.585
Drinking history (yes/no)	2/6	18/136	0.567
HBV DNA level (>10^5^/≤10^5^)	5/3	102/52	1.000
HS (+/-)	4/4	73/81	1.00
Inflammation, n (%)			0.219
G0	0	1(0.6%)	
G1	1(12.5%)	60(39.0%)	
G2	5(62.5%)	61(39.6%)	
G3	2(25%)	29(18.8%)	
G4	0(0)	3(2.0%)	
Fibrosis, n (%)			
S0	0	12 (7.8%)	0.006
S1	0	65 (42.2%)	
S2	4 (50%)	46 (29.9%)	
S3	4 (50%)	31 (20.1%)	

BMI, body mass index; HBV DNA, hepatitis B virus deoxyribonucleic acid

### OA promotes cell proliferation and migration

**Figure 4:**
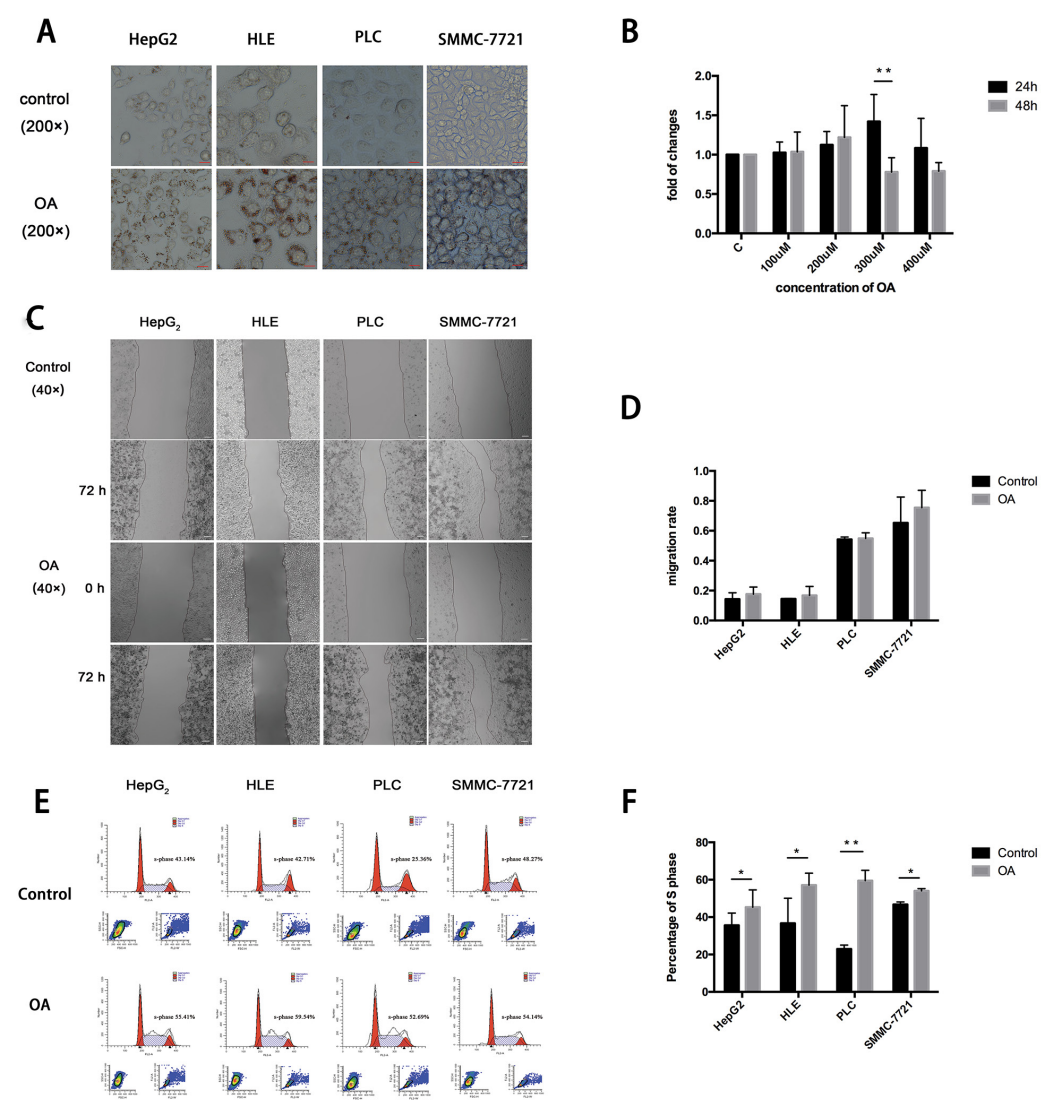
OA promotes HCC cells proliferation, migration **(A)** Oil red O staining shows lipid accumulation in HepG_2_, HLE, PLC and SMMC-7721 cells. **(B)** CCK-8 assay shows OA promotes the proliferation of HepG_2_ cell at a final concentration of 300 μM for 24 h. **(C)** Scratch assay of HCC cells. **(D)** Statistics of migration rate. (D-E) Flow cytometer analyses shows S phase of cells treated with OA was higher. Each test was repeated in three times (n=3). *P < 0.05 and **P < 0.01. Scale bars: 10 μm (A); 100 μm (C).

The HCC cells steatosis model was successfully established using OA (Figure 4A). The CCK-8 assay showed that proliferation of HepG_2_ cell was promoted significantly after 24 h at a final concentration of 300 μM (Figure 4B). The scratch assay showed that the migration of four kinds of HCC cells were promoted by OA, but yet reaching significant levels (Figure 4C-4D). Flow cytometer analyses found that cell cycle analyses showed that the S phase was higher in the cells exposed to OA than those without treatment (Figure 4E-4F). The experiments were repeated for three times.

## DISCUSSION

With the increasing incidence of NAFLD, the existence of concomitant hepatitis B and fatty liver is becoming more common in clinical practice. A study of the prognosis, treatment outcomes, and possible underlying mechanisms by Zheng et al reported that BMI, waist-to-hip ratio, fasting blood glucose, serum triglyceride levels, and serum total cholesterol appeared to be influencing factors of CHB combined with HS. HS in CHB patients was reported to be closely related to changes in anthropometric indices and metabolic factors, but not HBV [13]. It has been widely recognized that the presence of HS accelerates progression of fibrosis in patients with chronic hepatitis C infection [14]. Nevertheless, whether HS is associated with the severity of fibrosis in CHB patients remains to be determined. A study of 5406 patients conducted in Taiwan found that fatty liver with HBV infection has a significant synergistic association with liver damage [15]. In the present study, however, hepatic steatosis had no significant impact on the development of cirrhosis in CHB patients. Our study has the strength of liver biopsy of all patients, which increased the accuracy of fibrosis at baseline. Liver biopsy is the current gold standard to assess hepatic inflammation and fibrosis in patients with CHB. However, it is difficult to repeat liver biopsy over a relatively short follow-up period; therefore, noninvasive markers of liver fibrosis are typically used in clinical practice. In this study, noninvasive marker, APRI, was applied to assess the degree of liver fibrosis. It has been reported that the annual incidence of cirrhosis in CHB patients is 0.7%–2.4%[16–18]. In this study, the cumulative probability of developing cirrhosis was not significant between patients with and without HS, thus HS was not an independent prognostic factor of disease progression by univariate analysis. The rate of progression to cirrhosis during the follow-up period was lower than previous reports because most patients with mild fibrosis are enrolled in this study. This result is likely explained by the small number of cases and the fluctuation of HBV DNA levels during the followed-up period.

The presence of HS is a common finding in CHB infection. However, it remains controversial whether HS influences the efficacy of antiviral therapy. A study carried out by Cindoruk et al. [19] showed that HS was not associated with viral load or treatment outcome in 140 patients with CHB infection. Ben et al. [20] also showed that HS does not seem to influence the progression of liver fibrosis or therapeutic response. However, Shi et al. [21] reported that treatment with polyethylene glycol IFN for 48 weeks had no influence on the virological response of CHB patients with HS, but did alter the biochemical response. Another study by Jin et al. [22] found that HS was significantly associated with failure of ETV treatment. The present study revealed that the HS did not seem to affect the outcome of antiviral therapy. However, the role of HS should be further investigated in prospective longitudinal clinical studies with larger patient populations.

The following features were found to be significant prognostic factors in CHB: the presence of bridging fibrosis, older age, persistence of HBV in serum, HBeAg carrier status, and HBV reactivation. In this study, univariate analysis showed that age and liver fibrosis stage, but not the presence of HS and HBV DNA level, were prognostic factors for the development of cirrhosis. Compared with their baseline, meanwhile, the APRI of both patients with HS and without HS had all decreased after antiviral therapy during the follow-up period. Therefore, the antiviral therapy may also delay the progress of liver fibrosis and cirrhosis.

There are some limitations in our study. One of the limitations is small sample size of patients in this longitudinal cohort despite a follow-up duration of 5 years. In order to address the shortcomings, *in vitro* cell culture model was used in research of NAFLD with CHB. Recently, OA-induced lipid accumulation in HepG_2_ cells may act as an *in vitro* model for studying NAFLD [23]. In this study, the HCC cells steatosis model was successfully established using OA. The biological behavior, proliferation and migration of HCC cells, were promoted *in vitro*. These results suggested that hepatic steatosis may aggravate liver disease, including the progression of cirrhosis and HCC.

## MATERIALS AND METHODS

### Patients

A total of 1510 CHB patients were confirmed by liver biopsy from March 2008 to March 2010 in Beijing You’an Hospital. Of these, 162 patients were prospectively followed-up for more than 5 years. According to the results of liver biopsy, the patients were divided into two groups: CHB without HS (n = 85) and CHB with HS (n = 77) (Figure 1). The inclusion criteria were (1) age of 18–65 years, (2) HBsAg positive for more than 6 months without antiviral therapy (3), liver biopsy conducted within 3 months before enrollment, and (4) informed consent was signed to participate in this study. The exclusion criteria of this cohort were: (1) evidence of alcohol addiction (defined as alcohol intake of ≥40g/day for men and ≥20g/day for women for a 5-year period, or alcohol intake of ≥0.80 g/day within 2 weeks); (2) coexisting infection with human immunodeficiency virus (HIV), hepatitis C virus, or other virus-related hepatitis; (3) poorly controlled diabetes (fasting glucose level of > 220 mg/dL); (4) heart failure with grade of III or IV, and (5) definite diagnosis of liver cirrhosis, HCC, or autoimmune or inherited metabolic liver disease. The study protocol was approved by the Beijing You’an Hospital Institutional Review Board and conducted in compliance with the Declaration of Helsinki. The informed consent was obtained from each patient.

### Clinical and laboratory data

The symptoms, signs, and components of metabolic syndrome (height, weight, and body mass index, BMI) were retrieved from medical records. A microparticle enzyme immunoassay was used to test for serum markers of hepatitis B (F. Hoffmann-La Roche AG, Basel, Switzerland). HBVDNA levels were measured using the COBAS® AmpliPrep/COBAS® TaqMan® quantitative measurement system (Roche Diagnostics Deutschland GmbH) that has a lower limit of serum HBVDNA detection of 20 IU/mL. Serum markers of human immunodeficiency virus were detected using an electrochemiluminescence immunoassay (Cobas 6000; Roche Diagnostics Deutschland GmbH). Liver function, such as alanine aminotransferase (ALT), aspartate aminotransferase (AST), renal function and serum lipids, were measured with an automatic biochemical analyzer (AU5400; Olympus Corporation, Tokyo, Japan). Blood parameters, including white blood cell (WBC) and platelet (PLT) counts, were measured using an automatic biochemical analyzer (XT-4000i; Sysmex Corporation, Kobe, Japan).

### Liver biopsy

Each patient was tested for platelet counts, bleeding time, clotting time, and prothrombin activity before undergoing liver biopsy. Liver biopsy was performed under ultrasound guidance with a disposable 18G biopsy gun and liver puncture needle. Liver tissue sample was conventionally formalin-fixed, paraffin-embedded, and sectioned into thicknesses of 4μm. Histological grading of inflammation (G0–G4) and staging of the liver fibrosis (S0–S4) were carried out according to Metavir classification criteria by a pathologist who was blinded to the clinical data. The degree of fibrosis was staged from 0 to IV (0, no fibrosis; I, mild; II, moderate; III, severe; IV, cirrhosis). In the classification of steatosis, less than 5% was considered normal, 6-33% as mild, 34-66% as moderate, greater than 67% as severe fat deposition [24].

### Definition of liver cirrhosis

Cirrhosis was defined as (1) histological conformation, (2) overt cirrhotic findings by ultrasonography or endoscopy, or (3) blood PLT level <100 × 10^9^/L and fibroscan >12.5KPa or AST-to-platelet ratio index (APRI) >2.

### APRI for noninvasive evaluation of liver fibrosis

APRI= (AST/ULN*) × 100/PLT [25]

Units: AST(U/L), PLT (10^9^/L), *ULN, upper limit of normal

APRI values of 0.50 or less was defined as no liver fibrosis. While APRI values of 2.00 or more was defined as liver cirrhosis [26].

### Definitions of antiviral response

Antiviral failure was defined as detection of resistance-associated mutation or occurrence of HBV DNA rebound (HBV DNA was detective in two consecutive tests following negative testing results) during nucleotide/nucleoside analogues (NAs) and/or pegylated interferon (IFN) therapy. Complete virological response (CVR) was defined as undetectable serum HBV DNA (lower limit of detection = 20 IU/mL) during the period of antiviral therapy.

### Follow-up and end-points

All patients had been prospectively followed-up for more than 5 years at outpatients or inpatients department. The end-point of follow-up was a diagnosis of cirrhosis or liver cancer, or death. The APRI as noninvasive markers was dynamically used to assess liver fibrosis progression.

### *In vitro* study

As the limitation of the short time of follow-up and the small number of case, we also evaluated the effect of steatosis induced using OA on the biological behavior of HCC cells. Human HCC cell lines (HepG_2_, HLE, PLC, and SMMC-7721) were kindly provided by professor DX Chen of Beijing Institute of Hepatology. HepG_2_, HLE, and PLC cells were cultured in Dulbecco's modified Eagle's medium (DMEM, Thermo Fisher Scientific, USA) supplemented with 10% fetal bovine serum (FBS, Sijiqing, China). SMMC-7721 cells were cultured in Roswell Park Memorial Institute (RPMI) 1640 (Thermo Fisher Scientific, USA) with 10% FBS. Four kinds of HCC cells were subcultured with 5 × 10^5^ cells/well at the 37 °C, 5% CO_2_ incubator. After 24 h incubation, the culture medium was removed and cells was fixed using 4% paraformaldehyde. Then, the cells were stained using oil red O (Sigma, diluted in double-distilled H_2_O 3:2) for 30 min at room temperature Lipid droplet accumulation was detected using microscopy.

Cell Counting Kit-8 (CCK-8, Dojindo Laboratories, Kumamoto, Japan) was used to assess cell proliferation. HepG_2_ cells were taken into 96-well plates with 5000 cells in each well. Different concentrations of OA were added into each well following 24 h or 48 h incubation. The absorbencies were measured at a wave length of 450 nm on a Universal Microplate Reader (EL x800). Cell proliferation was calculated in term of the formula: cell proliferation%= [(A570 or 450sample-background)/ (A570 or 450 control-background)] ×100%. HCC cells migration was assessed using scratch assay. Four kinds of HCC cell lines were pipetted into 6-well microplates with 5 × 10^5^ cells in each well. After completely attachment, the cells were treated using different concentrations of OA. A scratch wound was made across the diameter of each plate using a sterile 10-μl pipette tip. Then, the cells were observed under microscopy at 0, 12, 24, 48, and 72h following treatment. Each test was repeated in three times.

Four kinds of HCC cell lines were cultured in serum-free medium for 24 h before treatment to arrest the cell cycle. Then, the serum-free medium was replaced by fresh DMEM containing 10% FBS. After treated with OA at a final concentration of 300 μM for 24h, the cells were stained by PI at a final concentration of 50 mg/L. DNA content was analyzed with a FACScan-420 flow cytometer (Becton-Dickinso, Germany). The distribution of cells in different cell cycle stages was determined according to the DNA content. Each test was repeated in three times.

### Statistical analysis

All statistical tests were performed using SPSS statistical software (version 17.0; IBM-SPSS, Inc., Chicago, IL, USA). Continuous variables are presented as means ± standard deviations and compared using the unpaired *t*-test and repeated measures analysis of variance, as appropriate. Categorical variables were compared using the chi-squared test. Progression analysis was performed using Kaplan–Meier survival curves and the log-rank test. Multivariate analysis was performed using logistic regression models. A two-sided probability (*p*) value of < 0.05 was considered statistically significant.

## Abbreviations

OA: oleic acid
HCC: hepatocellular carcinoma
AST: aspartate aminotransferase
APRI: AST-to-platelet ratio index
CHB: chronic hepatitis B
NAFLD: nonalcoholic fatty liver disease
HS: hepatic steatosis
NASH: nonalcoholic steatohepatitis
BMI: Body mass index
NAs: nucleotide/nucleoside analogues
HBV DNA: hepatitis B virus deoxyribonucleic acid
CCK-8: Cell Counting Kit-8
ETV: entecavir
ADV: adefovir
LAM: lamivudine
LDT: telbivudine
CVR: Complete virological response
HBeAg: hepatitis B e antigen
WBC: white blood cell
